# Involvement of inhibitory PAS domain protein in neuronal cell death in Parkinson’s disease

**DOI:** 10.1038/cddiscovery.2015.15

**Published:** 2015-08-17

**Authors:** S Torii, S Kasai, A Suzuki, Y Todoroki, K Yokozawa, K-I Yasumoto, N Seike, H Kiyonari, Y Mukumoto, A Kakita, K Sogawa

**Affiliations:** 1 Department of Biomolecular Sciences, Graduate School of Life Sciences, Tohoku University, Sendai, Japan; 2 Department of Pathology, Brain Research Institute, University of Niigata, Niigata, Japan; 3 Animal Resource Development Unit, RIKEN Center for Life Science Technologies, Kobe, Japan; 4 Genetic Engineering Team, RIKEN Center for Life Science Technologies, Kobe, Japan

## Abstract

Inhibitory PAS domain protein (IPAS), a repressor of hypoxia-inducible factor-dependent transcription under hypoxia, was found to exert pro-apoptotic activity in oxidative stress-induced cell death. However, physiological and pathological processes associated with this activity are not known. Here we show that IPAS is a key molecule involved in neuronal cell death in Parkinson’s disease (PD). IPAS was ubiquitinated by Parkin for proteasomal degradation following carbonyl cyanide *m*-chlorophenyl hydrazone treatment. Phosphorylation of IPAS at Thr12 by PTEN-induced putative kinase 1 (PINK1) was required for ubiquitination to occur. Activation of the PINK1–Parkin pathway attenuated IPAS-dependent apoptosis. IPAS was markedly induced in the midbrain following 1-methyl-4-phenyl-1,2,3,6-tetrahydropyridine (MPTP) administration, and IPAS-deficient mice showed resistance to MPTP-induced degeneration of dopaminergic neurons in the substantia nigra pars compacta (SNpc). A significant increase in IPAS expression was found in SNpc neurons in patients with sporadic PD. These results indicate a mechanism of neurodegeneration in PD.

## Introduction

Parkinson’s disease (PD) is a progressive neurodegenerative disorder that affects the control of body movements.^1,2^ The motor symptoms of the disease are associated with the death of dopaminergic neurons in the substantia nigra pars compacta (SNpc). The majority of PD cases are sporadic, accounting for 90–95% of cases. The pathogenesis of sporadic PD has yet to be established, but it is suggested that genetic predisposition and environmental toxins causing mitochondrial dysfunction and oxidative stress may be involved.^3^ There are a number of neurotoxins that may cause PD.^4^ Of these toxins, 1-methyl-4-phenyl-1,2,3,6-tetrahydropyridine (MPTP) may inhibit mitochondrial complex I after transformation to 1-methyl-4-phenylpyridinium (MPP^+^), resulting in an increase in reactive oxygen species (ROS) generation. Examination of the mechanism of cell killing by MPTP demonstrated that the major mechanism is Bax-dependent apoptosis.^5^

The remaining 5–10% PD cases are caused by genetic mutations and loci with causative mutations in six genes and a number of additional unidentified genes have been associated with autosomal dominant or recessive PD.^6^ The autosomal recessive mutations in the Parkin gene (*PARK 2*) that encode an E3 ubiquitin ligase are the most common cause of early onset PD. Recently, *in vitro* studies have shown that Parkin can mediate autophagy of damaged mitochondria (termed mitophagy) downstream of the serine/threonine-protein kinase PTEN-induced putative kinase 1 (PINK1)^7^ through ubiquitination of mitochondrial membrane proteins.^8,9^ These observations demonstrate that PINK1 and Parkin may mediate a pathway of mitophagy for mitochondrial quality control. The most straightforward mechanism by which the recessive loss of Parkin could cause apoptosis of dopaminergic neurons would include accumulation of neurotoxic substrate proteins in the neurons. Along this line, substrates of Parkin have been investigated and a number of Parkin substrates that could affect neuronal cell death in PD pathogenesis have been reported.^10,11^

It is unclear whether there is a common mechanism between cell death invoked by genetic and environmental factors for selective loss of dopaminergic neurons in the SNpc of PD patients. However, accumulating evidence that mutations and single-nucleotide polymorphisms in the PARK genes may contribute to the etiology of sporadic PD suggests the presence of a common mechanism.^10^ Considering the recently found close connection between PARK genes and mitochondrial quality control, some association between ROS from impaired mitochondria and Parkin substrates may be expected. However, studies that suggest a mechanistic link between accumulation of substrates and environmental factors have rarely been performed. Furthermore, involvement of ROS in the pathways associated with this accumulation is largely unknown.

Inhibitory PAS domain protein (IPAS) is one of splice variants of hypoxia-inducible factor (HIF)-3*α*,^12^ and was initially characterized as a repressor for hypoxic gene activation by HIF-1.^13^ Recently, we found that IPAS gene expression was also upregulated by the activation of NF-*κ*B by CoCl_2_-induced ROS^14^ or tumor necrosis factor-*α* ^15^ in rat pheochromocytoma PC12 cells. Furthermore, we found that IPAS acts as a pro-apoptotic protein on the mitochondria with properties similar to BH3-only proteins in CoCl_2_-induced cell death in PC12 cells.^16^ The mechanism underlying IPAS-induced apoptosis included recruitment of Bax to mitochondria and liberation of Bax by neutralizing anti-apoptotic members such as Bcl-x_L_. This ultimately leads to cytochrome c release and activation of caspase-3.

In this paper, we describe that mitochondrial IPAS was subjected to carbonyl cyanide *m*-chlorophenyl hydrazone (CCCP)-dependent phosphorylation and following ubiquitination by PINK1 and Parkin, respectively, resulting in attenuation of apoptosis. IPAS-deficient mice were resistant to neuronal cell death induced by MPTP. Finally, we present evidence for enhanced expression of IPAS protein in SNpc neurons of postmortem brain samples from sporadic PD patients.

## Results

### Binding of IPAS to Parkin

**Figure 1:**
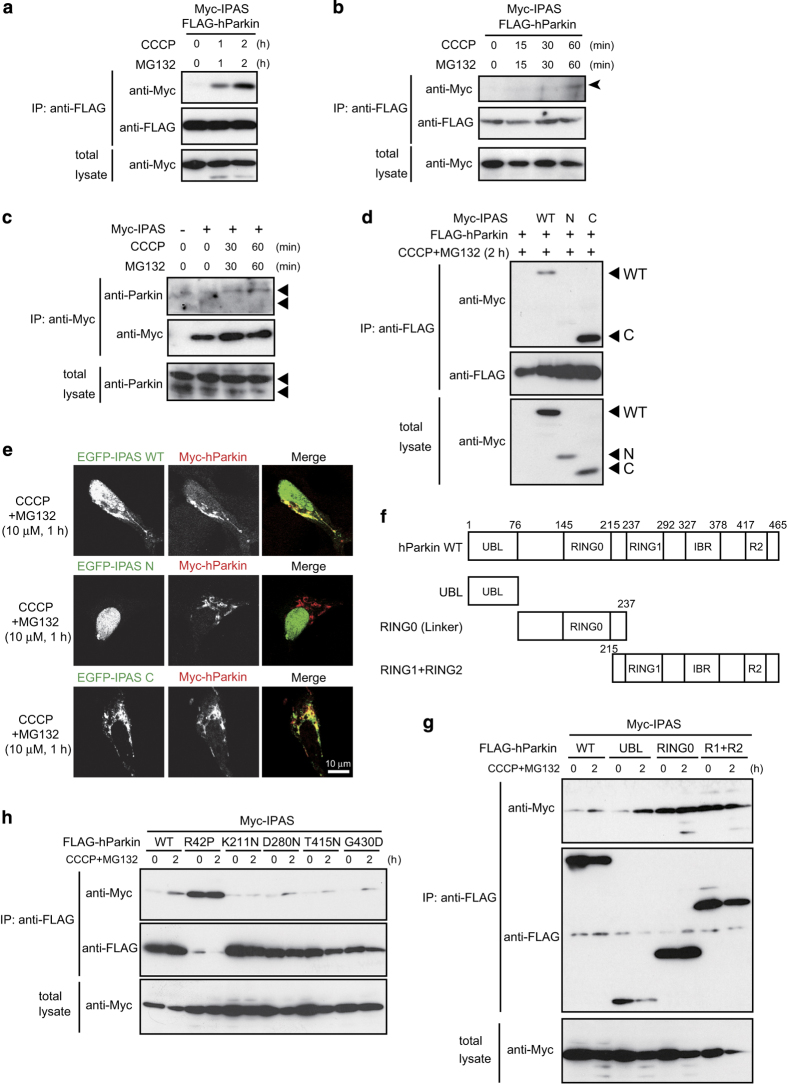
Binding of IPAS to Parkin. (**a** and **b**) Induction of IPAS binding to Parkin by CCCP and MG132 treatment. HeLa cells (**a**) and SH-SY5Y cells (**b**) were transfected with expression plasmids encoding Myc-IPAS and FLAG-hParkin for 24 h, treated with CCCP (10 *μ*M) and MG132 (10 *μ*M), and analyzed by immunoprecipitation with antibody against FLAG followed by immunoblotting with antibody against Myc. (**c**) Binding of IPAS to endogenous hParkin. SH-SY5Y cells were transfected with pMyc-IPAS, and treated as in **b**. Immunoblotting was performed using anti-Parkin antibody. Arrowheads indicate two bands of Parkin. (**d**) Binding of IPAS C-terminal region to Parkin. HeLa cells were transfected with deletion plasmids of IPAS together with pFLAG-hParkin. Co-IP assays were carried out as in **a**. (**e**) Colocalization of IPAS WT and IPAS C with Parkin. SH-SY5Y cells were transfected with pEGFP-IPAS WT, pEGFP-IPAS N or pEGFP-IPAS C together with pMyc-hParkin, and treated with CCCP and MG132. Localization of IPAS WT, IPAS deletions and Parkin was observed using a confocal microscope. (**f**) Schematic representation of structure of Parkin and its deletion mutants. (**g** and **h**) Binding of IPAS to Parkin with deletions (**g**) or pathogenic point mutations (**h**). HeLa cells were transfected with pMyc-IPAS and plasmids for Parkin mutants. Co-IP assays were carried out as in **a**. UBL, ubiquitin like domain; RING0, RING0 domain; RING1, RING1 domain; IBR, In-Between-RING fingers domain; R2, RING2 domain.

To investigate the binding of IPAS to human Parkin (hParkin), co-immunoprecipitation (Co-IP) experiments were performed in SH-SY5Y cells, which express functional Parkin, and HeLa cells, which do not.^17^ Binding was not observed in untreated HeLa cells, but was induced by treatment with CCCP and MG132 (Figure 1a). Treatment with CCCP or MG132 alone could induce very weak or no interaction, respectively, between IPAS and Parkin (Supplementary Figures S1a and b). Similar results were obtained in SH-SY5Y cells (Figure 1b). IPAS also bound to mouse Parkin (mParkin) in a similar fashion in both cells (Supplementary Figure S1c). Furthermore, we examined whether endogenous hParkin could bind to IPAS. Figure 1c shows that binding of IPAS to the major form of endogenous hParkin was induced by treatment with CCCP and MG132.

Co-IP assays using two IPAS deletion mutants, Myc-IPAS N and Myc-IPAS C (structures shown in Supplementary Figure S2a), revealed that hParkin and mParkin bound IPAS C but not IPAS N (Figure 1d and Supplementary Figure S2b). To confirm this result, we investigated colocalization of the IPAS deletion mutants and hParkin or mParkin using confocal microscopy (Figure 1e and Supplementary Figure S2c). hParkin translocated to mitochondria in response to CCCP treatment as previously reported.^18^ Although CCCP treatment did not significantly affect subcellular localization of IPAS wild type (WT) and its deletion mutants (data not shown), IPAS WT and IPAS C were colocalized on the mitochondria with hParkin and mParkin following CCCP and MG132 treatment. We expressed hParkin deletion mutants (Figure 1f) to examine the region necessary to bind IPAS. The UBL region bound to IPAS in a CCCP and MG132-dependent manner. However, the RING0 and RING1+RING2 regions bound to IPAS with enhanced affinity, regardless of CCCP and MG132 treatment (Figure 1g). These results suggest that multiple regions are involved in the binding to IPAS, and that the UBL region might be responsible for CCCP and MG132-dependent binding. Moreover, we investigated binding of IPAS to pathogenic mutants of hParkin (Figure 1h). We chose the R42P, K211N, D280N, T415N and G430D missense mutations localized in the UBL, RING0, RING1, IBR and RING2 regions, respectively. The R42P mutant bound to IPAS with higher affinity than the WT, regardless of CCCP and MG132 treatment. Conversely, binding of the other pathogenic mutants to IPAS was weak when compared with that of WT.

### Polyubiquitination of IPAS by Parkin

**Figure 2:**
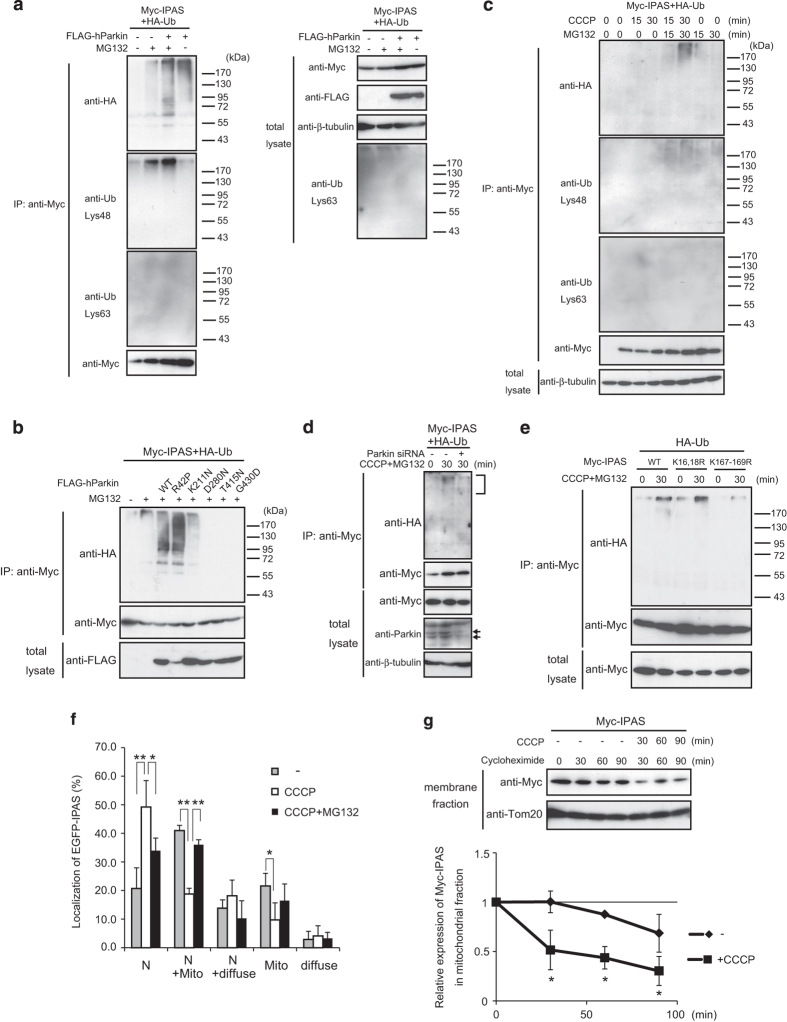
Polyubiquitination of IPAS by Parkin. (**a**) Polyubiquitination of IPAS by Parkin through K48 linkage. HeLa cells were transfected with indicated combinations of plasmids, treated with or without MG132, and analyzed by immunoprecipitation with antibody against Myc followed by immunoblotting with antibody against HA, Ub-Lys48 or Ub-Lys63. (**b**) E3 ligase activity of Parkin mutants towards IPAS. HeLa cells were transfected with FLAG-tagged WT or mutated Parkin plasmid, and analyzed as in **a**. (**c**) Polyubiquitination of IPAS by endogenous Parkin. SH-SY5Y cells were transfected with pMyc-IPAS and pHA-Ub, treated with CCCP and/or MG132 for the indicated times, and analyzed as in **a**.(**d**) Decrease in polyubiquitination of IPAS by Parkin siRNA treatment. Parkin siRNA treated (24 h) SH-SY5Y cells were transfected for 24 h with pMyc-IPAS and pHA-Ub. Cells were treated with CCCP and MG132 for 30 min, and analyzed as in **a**. (**e**) Ubiquitinated lysine residues in IPAS. Myc-IPAS K16,18R and K167–169R mutants were expressed in SH-SY5Y cells and analyzed their ubiquitination as in **a**. (**f**) Decrease in mitochondrial localization of IPAS by CCCP treatment. SH-SY5Y cells were transfected with pEGFP-IPAS, and treated with CCCP or CCCP plus MG132. One hour after the treatment, cells that indicated subcellular localization of EGFP-IPAS were counted. *n*=3, *P<0.05, **P<0.01. (**g**) Increased degradation of mitochondrial IPAS by CCCP treatment. SH-SY5Y cells were transfected with pMyc-IPAS and treated with CCCP in the presence of cycloheximide (20 *μ*g/ml). Cells were collected at the indicated times, and membrane fractions containing mitochondrial membranes (Supplementary Figure S3a) were analyzed by immunoblotting using antibodies against Myc and Tom20 (upper panel). Myc-IPAS and Tom20 levels in each sample were determined by image analysis using ImageJ software (National Institutes of Health, Bethesda, MD, USA). Ratios of Myc-IPAS to Tom20 were determined from three independent experiments. *P<0.05. N, nuclear localization; Mito, mitochondrial localization; diffuse, diffuse cytoplasmic localization.

We investigated ubiquitination of IPAS by Parkin in HeLa cells, and found that polyubiquitination of IPAS clearly occurred in response to MG132 treatment (Figure 2a, lane 3). IPAS was weakly ubiquitinated without overexpression of FLAG-hParkin (Figure 2a, lane 2), suggesting that an E3 ubiquitin ligase(s) other than Parkin was involved in the basal level of ubiquitination of IPAS. Polyubiquitin chains were linked through the Lys48 residue of ubiquitin, which is most commonly associated with proteins targeted for proteasomal degradation. Similar results were obtained when FLAG-mParkin was used instead of FLAG-hParkin (Supplementary Figure S1d). The five pathogenic mutants of hParkin used in the binding experiments were expressed in HeLa cells and their ubiquitination activity toward IPAS was investigated (Figure 2b). The Parkin R42P mutant induced higher levels of ubiquitinated IPAS compared with WT hParkin. The K211N mutant showed a very weak activity, and D280N, T415N and G430D mutants showed no activity toward IPAS. Lower levels of ubiquitination than control by the D280N, T415N and G430D mutants might arise from their dominant negative effect.

Next, we examined whether endogenous hParkin could ubiquitinate IPAS in response to mitochondrial depolarization. Figure 2c shows that K48-linked polyubiquitination of IPAS was notable 30 min after treatment with CCCP and MG132. Knockdown of endogenous hParkin by siRNA treatment decreased IPAS polyubiquitination (Figure 2d). We mutated lysine residues to arginine in the two lysine clusters (amino acids 16–18 and 167–169) of IPAS to determine polyubiquitination sites. IPAS K16–18R mutant was polyubiquitinated at a level similar to WT (Figure 2e). However, polyubiquitination of the K167–169R mutant was greatly decreased, suggesting that at least one lysine residue in the cluster may be ubiquitinated by hParkin.

When EGFP-IPAS was expressed in SH-SY5Y cells, various localization patterns of EGFP-IPAS were observed (Figure 2f, Torii *et al.* ^16^). CCCP treatment reduced the ratio of cells displaying mitochondrial localization of IPAS to ~45% of untreated control (Figure 2f, N+Mito and Mito patterns). This reduction was abrogated by co-treatment with MG132. We next examined the degradation rate of IPAS on mitochondria with or without CCCP treatment (Figure 2g) using membrane fraction (Supplementary Figure S3a). Membrane-bound Myc-IPAS rapidly decreased to ~25% 90 min after CCCP treatment, whereas it remained at >70% in untreated cells.

### Binding of IPAS to PINK1 in mitochondria

**Figure 3:**
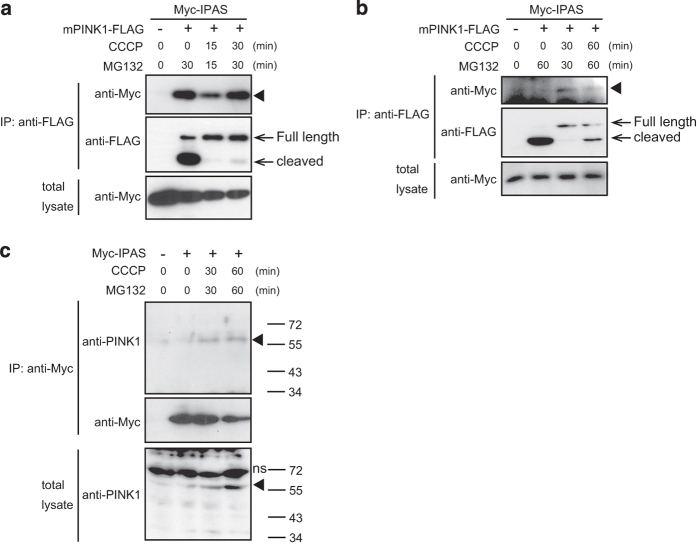
Binding of IPAS to PINK1. (**a** and **b**) Binding of IPAS to PINK1. HeLa cells (**a**) and SH-SY5Y cells (**b**) were transfected with pMyc-IPAS and pPINK1-FLAG, and membrane fractions were isolated. Immunoprecipitation was performed with antibody against FLAG followed by immunoblotting with antibody against Myc. (**c**) Binding of IPAS to endogenous PINK1. SH-SY5Y cells were transfected with pMyc-IPAS and treated with CCCP and MG132 for indicated time periods. After solubilization of membrane fractions, immunoprecipitation was performed using anti-Myc antibody followed by immunoblotting with antibody against PINK1. ns, nonspecific band.

We investigated the involvement of PINK1 in the Parkin-dependent degradation of IPAS. As reported, accumulation of full-length and cleaved fragments of PINK1 was observed in the mitochondria after treatment with CCCP and/or MG132 (Supplementary Figures S3b and c, and Jin *et al.* ^19^). Co-IP experiments clearly showed that IPAS could bind mitochondrial PINK1 following CCCP and/or MG132 treatments (Figures 3a and b). Endogenous PINK1 also interacted with overexpressed Myc-IPAS (Figure 3c). Immunostaining experiments showed that PINK1-Myc was colocalized with EGFP-IPAS WT and EGFP-IPAS C on mitochondria following treatment with CCCP and MG132 (Supplementary Figure S4).

### Phosphorylation of IPAS by PINK1

**Figure 4:**
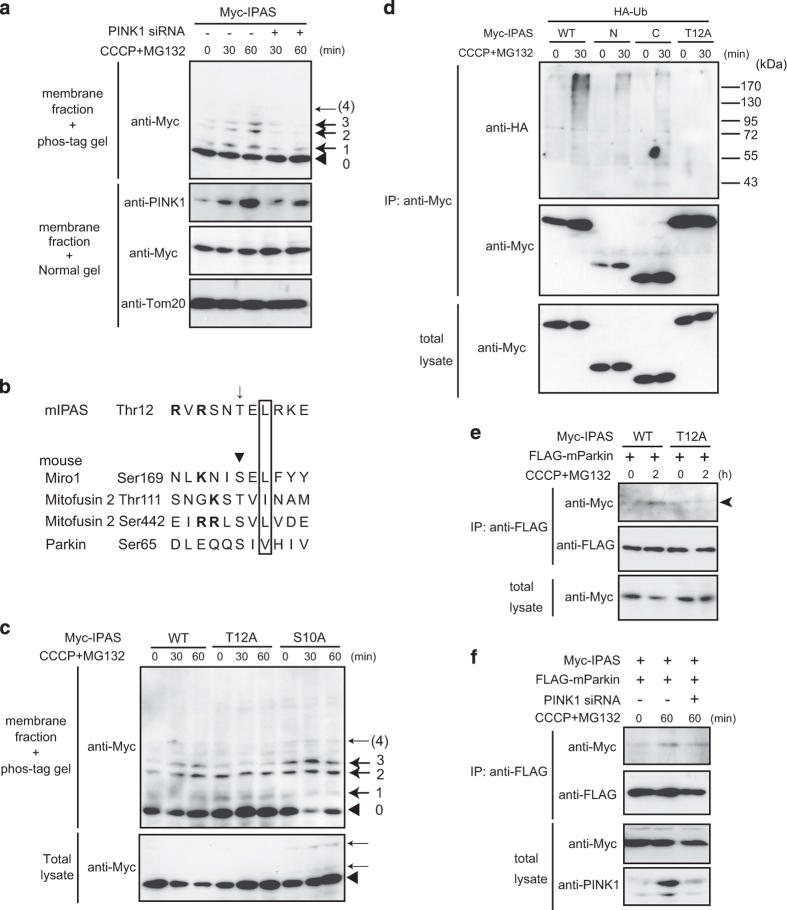
Phosphorylation of IPAS by PINK1. (**a**) Phos-tag-based immunoblot analysis of CCCP-activated IPAS. PINK1 siRNA treated (24 h) SH-SY5Y cells were transfected with pMyc-IPAS for 24 h and treated with CCCP and MG132 as indicated, and membrane fractions were analyzed by Phos-tag immunoblotting using anti-Myc antibody. Band 0 indicates unphosphorylated IPAS. Bands 1, 2, 3 and 4 indicate phosphorylated IPAS. (**b**) Alignment of amino acid sequences containing the putative phosphorylation sites of IPAS and phosphorylation sites of several PINK1 substrates. The putative phosphorylated Thr residue and known phosphorylated Ser or Thr residues are indicated by an arrow and arrowhead, respectively. Hydrophobic amino acids found two residues after phosphorylation sites are boxed, and basic amino acids are designated in bold. (**c**) Phosphorylation of Thr12 by PINK1. SH-SY5Y cells were transfected with pMyc-IPAS WT, T12A or S10A, and analyzed by Phos-tag immunoblotting as in **a**. (**d**) Lack of polyubiquitination of IPAS T12A mutant by Parkin. SH-SY5Y cells were transfected for 24 h with plasmids for IPAS WT, IPAS N, IPAS C or IPAS T12A together with HA-Ub, treated with CCCP and MG132 for 30 min and analyzed by immunoblotting with antibody against HA. (**e**) Lack of binding of IPAS T12A mutant to Parkin. SH-SY5Y cells were transfected with plasmids of IPAS WT or the T12A mutant, and treated with CCCP and MG132 for 2 h. Immunoprecipitation was performed with antibody against FLAG followed by immunoblotting with antibody against Myc. (**f**) Attenuation of IPAS binding to Parkin by PINK1 siRNA treatment. PINK1 siRNA treated SH-SY5Y cells were transfected with pMyc-IPAS and FLAG-Parkin and treated with CCCP and MG132 for 60 min. Immunoprecipitation was performed as in **e.**

Because IPAS bound to PINK1 on mitochondria, we analyzed whether IPAS was a substrate of PINK1 kinase using Phos-tag SDS-PAGE. Three mobility-shifted bands (Figure 4a, bands 1–3) were clearly induced by treatment with CCCP and MG132. Treatment with PINK1 siRNA strongly decreased the intensity of all the bands. Notably, the intensities of bands 2 and 3 decreased to the basal level. These results indicate that multiple sites of IPAS were phosphorylated by PINK1. Referring to previously reported phosphorylation sites in Miro, Mitofusin 2 and Parkin by PINK1, a possible phosphorylation site (Thr12) of IPAS was identified (Figure 4b) and mutated to Ala. When the phosphorylation status of IPAS T12A mutant was analyzed by Phos-tag SDS-PAGE, the intensity of band 3 was significantly and selectively reduced (Figure 4c). The IPAS S10A mutant used as a control showed a pattern of phosphorylation similar to that of WT IPAS. The presence of a weak residual intensity of band 3 found in the T12A mutant may suggest the presence of a constitutively phosphorylated moiety of IPAS that co-migrates with the Thr12-phosphorylated IPAS. We examined polyubiquitination of the IPAS T12A mutant by endogenous hParkin (Figure 4d). Surprisingly, ubiquitination of the T12A mutant was completely lost, although IPAS C and IPAS N were ubiquitinated at low levels. We further examined whether IPAS T12A binds to mParkin. Figure 4e shows that the interaction decreased to a basal level regardless of CCCP and MG132 treatment. Furthermore, PINK1 siRNA treatment decreased the interaction between IPAS and mParkin (Figure 4f).

### Attenuation of IPAS-dependent apoptosis by activation of PINK1–Parkin pathway

**Figure 5:**
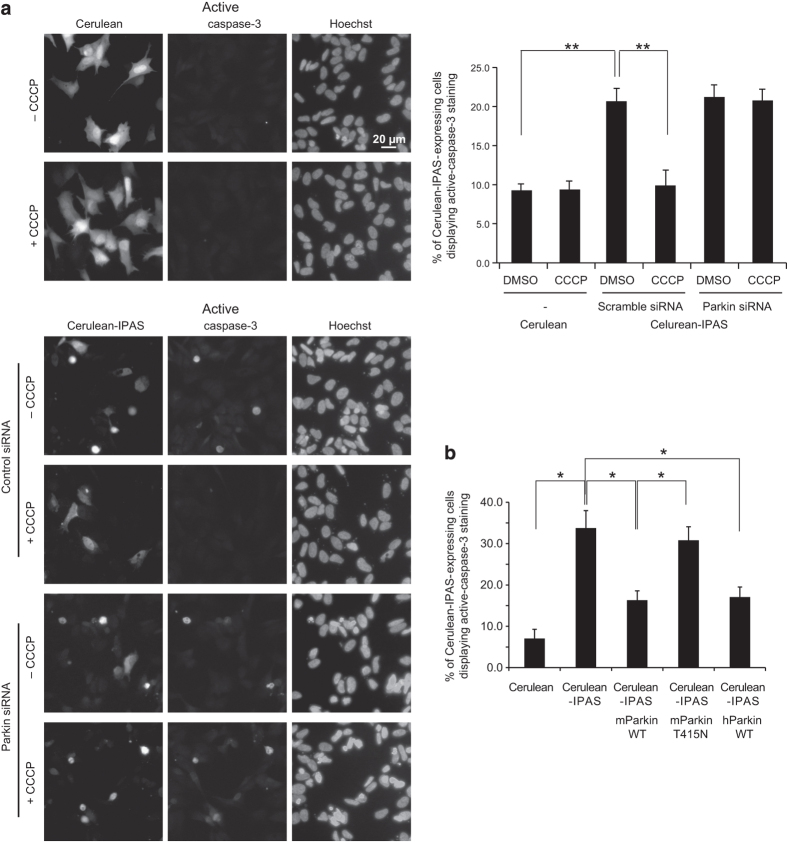
Attenuation of IPAS-induced apoptosis by the activation of the PINK1–Parkin pathway. (**a**) Attenuation of IPAS-induced apoptosis by CCCP treatment. Parkin siRNA treated (24 h) SH-SY5Y cells were transfected with pCerulean-IPAS. Four hours after transfection, cells were treated with CCCP (20 *μ*M) for 100 min, and cultured for 24 h. Cells were stained with active caspase-3 antibody and Hoechst 33342, and were observed using a fluorescence microscope. A minimum of 300 transfected cells per sample was counted and ratio of active caspase-3 positive cells to Cerulean-IPAS-expressing cells is calculated (right). Data shown in bar graphs are mean±S.D. of three independent experiments. **P<0.01 for indicated comparison. (**b**) SH-SY5Y cells were transfected with pCerulean, pCerulean-IPAS, pCerulean-IPAS+pMyc-mParkin WT, pCerulean-IPAS+pMyc mParkin T415N or pCerulean-IPAS+pMyc-hParkin, stained as in **a**, and observed using a fluorescence microscope. Representative images of transfected cells are shown in Supplementary Figure S5. A minimum of 100 transfected cells per sample were processed as in **a**. Data shown in bar graphs are mean±S.D. of three independent experiments. *P<0.05.

We investigated whether activation of the PINK1–Parkin pathway protects cells from IPAS-induced apoptosis. Overexpression of Cerulean-IPAS in SH-5YSY cells induced activation of caspase-3 in ~21% of Cerulean-IPAS-expressing cells (Figure 5a). When cells were treated with CCCP, the number of cells with active caspase-3 decreased to the basal level. Parkin siRNA treatment abolished the anti-apoptotic activity of CCCP. Furthermore, we overexpressed mParkin and hParkin together with Cerulean-IPAS (Figure 5b and Supplementary Figure S5). Parkin could inhibit activation of caspase-3 in IPAS-expressing cells, probably because of the increased basal level of Parkin on mitochondria. Conversely, the Parkin T415N mutant (a catalytically inactive mutant), showed no anti-apoptotic activity.

### Induction of IPAS in the midbrain of MPTP-treated mice

**Figure 6:**
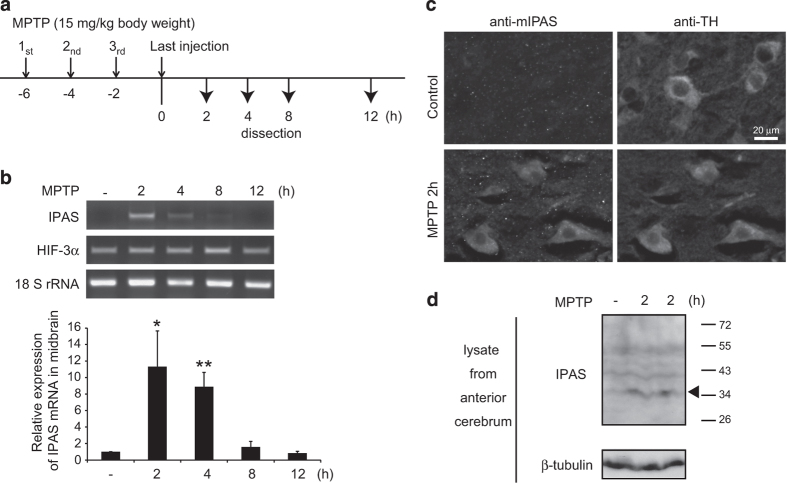
Induction of IPAS by MPTP administration in mouse brains. (**a**) Schedule of MPTP administration. Small arrows indicate time points of MPTP administration, and total RNA was prepared from midbrains at time points shown by large arrows. (**b**) Induction of IPAS mRNA in the midbrain by intraperitoneal injection of MPTP. IPAS mRNA expression levels in the midbrain at indicated time points were determined by RT-PCR. PCR for IPAS and HIF-3*α* was performed using primers recognizing IPAS-specific exons 1a and 4a and HIF-3*α*-specific exons 12 and 15, respectively. The PCR products were analyzed on a 2% agarose gel. Representative images of three independent experiments were shown (upper). Each band was quantified by using ImageJ software (National Institutes of Health). Data shown in bar graphs are mean±S.D. of three independent experiments (lower). *P<0.05, **P<0.01. (**c**) Induction of IPAS protein in the TH-positive neurons by MPTP. Brain sections were prepared from mice 2 h after final injection of MPTP. Neurons in the SNpc were stained with anti-TH and anti-IPAS antibodies. (**d**) Induction of IPAS protein by MPTP. Tissue lysates of cerebrum from one control and two MPTP-treated mice were prepared 2 h after final MPTP injection and analyzed by immunoblotting using anti-IPAS antibody. An arrowhead shows the band of IPAS.

We examined induction of IPAS in the midbrain of male C57BL/6J mice that received four intraperitoneal injections of MPTP (15 mg/kg) at 2**-**h intervals (Figure 6a). IPAS mRNA, but not HIF-3*α* mRNA, was strongly induced 2–4 h after final injection of MPTP, and the elevated level returned to the basal level within 12 h (Figure 6b). Similar levels of IPAS induction were observed in the cerebrum and cerebellum (Supplementary Figure S6a), suggesting that induction of IPAS by MPTP occurred throughout the whole brain. Immunohistochemical analysis revealed that IPAS protein was expressed and localized in the cytoplasm of tyrosine hydroxylase (TH)-positive neurons in the MPTP-treated SNpc (Figure 6c). IPAS was also expressed in normoxic Purkinje cells of the cerebellum (Supplementary Figure S6b), and the expression was enhanced in response to hypoxia as described by Makino *et al.* ^13^ Immunoblot analysis showed that IPAS protein was induced by MPTP, and had a molecular mass of ~34 kDa (Figure 6d), in good agreement with the calculated molecular mass of IPAS.

### Attenuation of MPTP-induced cell death in the SNpc of IPAS-deficient mice

**Figure 7:**
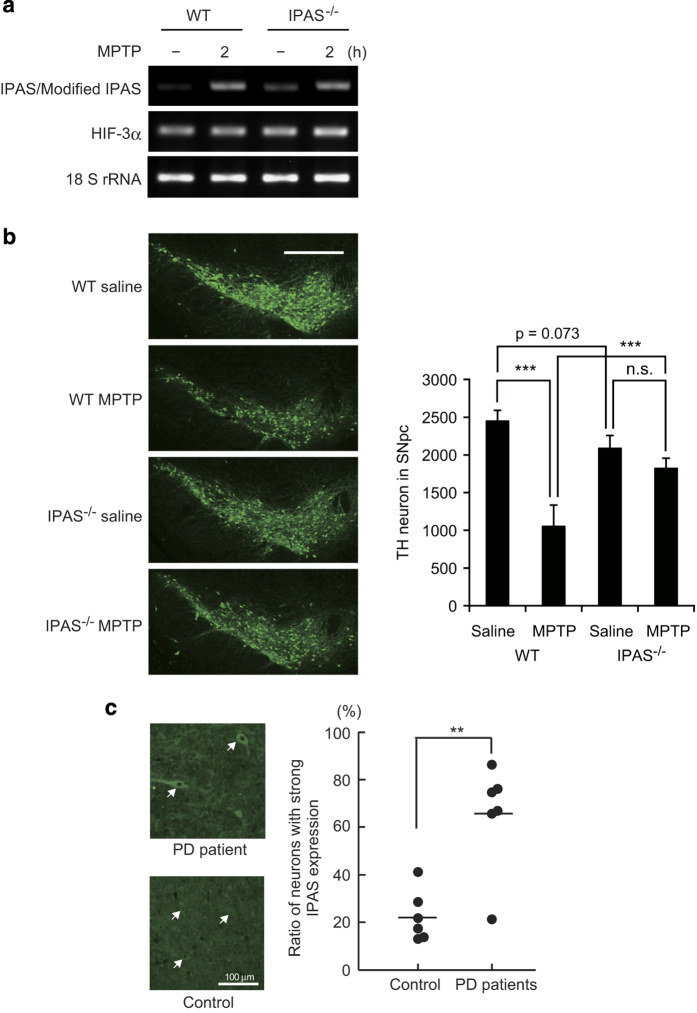
Attenuation of MPTP-induced neuronal cell death in IPAS-deficient mice and IPAS expression in human SNpc. (**a**) Expression of HIF-3*α* mRNA in IPAS-deficient mice. IPAS-deficient mice and WT littermates were treated with MPTP or saline according to the procedure shown in Figure 6a, and total RNA was extracted 2 h after final injection. Expression of HIF-3*α* and IPAS mRNA in the midbrain was analyzed by RT-PCR as described in Figure 6b. (**b**) Decreased cell loss of TH-positive neurons in the SNpc of IPAS-deficient mice treated with MPTP. Immunofluorescence analysis was performed using coronal sections through midbrains of IPAS-deficient mice and WT littermates administered with saline (*n*=5) or MPTP (*n*=4). Every third section of each brain was immunostained for TH and observed with a fluorescence microscope. Representative images were shown (left). scale bar, 500 *μ*m. Number of TH-positive neurons in each SNpc was scored (right). ***P<0.001 for indicated comparison. (**c**) Enhanced expression of IPAS in the neurons of the SNpc from PD patients. Immunostaining of SNpc from PD patients and normal controls with an anti-human IPAS antibody. Representative images are shown left. Arrows show neurons with IPAS expression. Cells with strong expression of IPAS in the neurons were counted using ImageJ software (National Institutes of Health), and the percentage of positive cells relative to the total number of neurons in each of PD patients (*n* = 6) and normal controls (*n* = 6) is indicated by a dot; the horizontal line represents the mean. **P<0.005. ns, not significant.

To investigate the causal relationship between IPAS expression and MPTP-induced cell death, we produced genetically modified mice lacking IPAS exon 16, which encodes the region essential for its pro-apoptotic activity.^16^ The structure of the targeting vector is shown in Supplementary Figure S7a, and homologous recombination was confirmed by genomic PCR and Southern blot analysis (Supplementary Figure S7b). Homozygous mutant mice were viable, fertile and born according to Mendelian ratios. IPAS^−/−^ mice had body weights comparable with WT littermates and displayed no obvious abnormalities. Male IPAS-deficient mice on C57BL/6 background (10–15-weeks old) were treated with MPTP. Modified IPAS mRNA was expressed in IPAS-deficient mice and induced by MPTP treatment (Figure 7a). HIF-3*α* mRNA levels were similar in IPAS-deficient mice and WT littermates. IPAS-deficient mice and control WT littermates were administered either MPTP (15 mg/kg) or an equal volume of saline according to the protocol shown in Figure 6a, and brains were analyzed 3 days after treatment. As expected, MPTP treatment substantially reduced the number of TH-positive neurons in the SNpc of WT littermates (Figure 7b). However, only a modest decrease in the number of TH-positive neurons after MPTP treatment was observed in the SNpc of IPAS-deficient mice. Interestingly, IPAS-deficient mice showed a tendency (*P* **=**0.073) to have fewer TH-positive neurons in the SNpc than WT littermates. The reason for this decrease is unknown. IPAS might be involved in the normal development of TH-positive neurons in the SNpc.

### IPAS expression in SNpc of PD patients

Formalin-fixed, paraffin-embedded sections of the midbrain of autopsied patients with sporadic PD and neurologically normal control individuals were analyzed by immunohistochemistry with an antibody specific for human IPAS. Clinical profiles of patients examined in this study are summarized in Supplementary Table S1. Neurons in the SNpc were distinguished from glial cells based on Hoechst staining, in which neuronal nuclei appear larger and less dense than glial nuclei. IPAS was expressed mainly in the cytoplasm of neurons, similar to the results observed in MPTP-induced mouse brains. The intensity of IPAS immunostaining was greater in the neurons of sporadic PD patients than in those of control individuals, although a weak-to-moderate expression of IPAS was also observed in the control individuals (Figure 7c).

## Discussion

PINK1-dependent phosphorylation of Parkin causes a conformational change that is necessary for Parkin translocation to mitochondria and the subsequent ubiquitination and degradation of mitochondrial proteins in mitophagy and mitochondrial dynamics.^20,21^ In this study, we showed that activated PINK1 also phosphorylates IPAS at several sites, including Thr12, which is prerequisite for recognition and ubiquitination of IPAS by Parkin. Like phosphorylation of Parkin by PINK1, this phosphorylation of IPAS might induce a conformational change that exposes the C-terminal binding region to Parkin. Similar masking of the C-terminal region of IPAS by its N-terminal region was previously observed in the CRM1 binding to the C-terminal leucine-rich nuclear export signal.^22^ Recently, Bcl-x_L_ was reported to be phosphorylated by PINK1, leading to impairment of the pro-apoptotic N-terminal cleavage of Bcl-x_L_.^23^ Thus, activation of the PINK1–Parkin pathway constitutes a stratified defense mechanism against apoptotic cell death, through degradation of pro-apoptotic IPAS and increased stabilization of anti-apoptotic Bcl-x_L_.

The PD-causative Parkin mutations K211N, T415N and G430D that inactivate E3 ubiquitin ligase activity attenuated binding to IPAS, and did not exhibit subsequent ubiquitination over controls (Figures 1h and 2b). A strong interaction of IPAS with the Parkin R42P mutant was found, and the mutation enhanced subsequent ubiquitination. Similar increased polyubiquitination by the R42P mutant was found in pathogenic Parkin substrate AIMP2.^24^ It was reported that Parkin modulates the assembly and activity of the 26S proteasome via its UBL domain, and the R42P mutation abrogates this function, suggesting a mechanism of cell death by the R42P mutation distinct from other pathogenic mutations.^25^ Another toxic property reported by Safadi and Shaw,^26^ and Schlehe *et al.* ^27^ was that the R42P mutation causes unfolding of the UBL domain, leading to its protein aggregation. The D280N mutation was shown to have weak autoubiquitination activity^20^ and to be recruited to the mitochondria in a manner similar to WT Parkin.^28^ This variant, however, failed to ubiquitinate IPAS (Figure 2b), although weak interaction with IPAS was observed. These findings regarding the interaction between IPAS and Parkin mutants strongly suggest that IPAS is a pathogenic substrate of PINK1 and Parkin. Consistent with this view, activation of PINK1 and Parkin by CCCP in SH-SY5Y cells antagonized IPAS-dependent apoptosis (Figure 5). A comprehensive study on the substrates of Parkin was performed by Sarraf *et al.*;^9^ however, IPAS was not found in the list of substrates. This is presumably due to lack of IPAS expression in the cultured cells used for the study.

Blockage of complex I of mitochondria by MPP^+^ leads to activation of Bax.^3^ However, other apoptotic factors involved in recruitment of Bax to mitochondria remain largely unknown. Induction of BH3-only protein Bim is postulated to be involved in Bax recruitment to mitochondria.^29^ The present study revealed that IPAS was induced acutely and transiently in TH-positive neurons by administration of MPTP. This temporal pattern of IPAS induction is in sharp contrast to that of Bim, which shows a low peak at around 24 h after administration. These observations suggest that IPAS and Bim might function cooperatively in the recruitment of Bax and neutralizing pro-survival proteins including Bcl-x_L_ in the neurodegenerative process. Strong induction of IPAS by MPTP was found not only in the midbrain but in other parts of the brain (Supplementary Figure S6), suggesting that selective cell death induced by MPTP in dopaminergic neurons in the SNpc cannot be fully attributed to IPAS activation. Cell type-specific post-translational modifications of IPAS may account for this selectivity and are thus currently under investigation.

Previously, we found that activation of IPAS transcription by oxidative stress was mediated via activation of the classical NF-*κ*B pathway.^14^ Activation of the NF-*κ*B pathway in dopaminergic neurons treated with MPTP was reported by Ghosh *et al.*,^30^ and we also confirmed activation of several NF-*κ*B target genes (data not shown). Recently, neural inflammation is considered to be involved in the pathogenesis of PD.^31^ Activated microglia may induce neural NF-*κ*B activation by releasing cytokines, which may lead to IPAS activation.^15^

For construction of IPAS-deficient mice, exon 16 (final exon), which is IPAS-specific and encodes the functional region of IPAS’s pro-apoptotic activity, is selected as the deleted region. This is to minimize effects on the expression of other splice variants. HIF-3*α* mRNA was detected in the IPAS-deficient mice at an expression level similar to WT littermates, suggesting that other splice variants can be expressed in IPAS-deficient mice. MPTP-induced cell loss was greatly attenuated in the IPAS-deficient mice. This finding demonstrates that MPTP-induced IPAS plays a key role in the MPTP-induced cell death of the dopaminergic neurons in the SNpc.

Recently emerging evidence suggests that HIF-1 expression slows progression of neurodegenerative diseases, including PD.^32^ Lee *et al.*^33^ reported that upregulation of HIF-*α* by inhibitors of HIF prolyl hydroxylases protects nigral dopaminergic cell loss in the SNpc of mice administered MPTP. Although various protection mechanisms were proposed, it could be at least partly explained that HIF-*α* induced by hypoxia or hypoxia-mimetic agents could sequester IPAS in the nucleus and prevent binding to mitochondrial Bcl-x_L_.

Increased IPAS expression was observed in SNpc neurons from sporadic PD patients compared with normal controls. This result is in accordance with the observation that NF-*κ*B is activated in the dopaminergic neurons of patients with PD.^34^ The weak expression of IPAS we found in midbrain tissues from normal control subjects might arise from aging-related NF-*κ*B activation. Age-dependent neuronal and glial activation of NF-*κ*B and its downstream genes in the hypothalamus and other regions was reported by Zhang *et al.*^35^ In addition to aging, we cannot strictly exclude the possibility that brain inflammation occurred in the control midbrains causing IPAS induction via activation of NF-*κ*B, although control brains were obtained from neurologically normal subjects.

In conclusion, our study strongly suggests that IPAS is critically involved in the pathogenesis of both sporadic and familial PD. The role of IPAS in the cell death of dopaminergic neurons does not exclude apoptotic contributions of other Parkin substrates analyzed thus far. The present study indicates that IPAS is a novel potential therapeutic target for sporadic and familial PD. Excessive ROS generation is suspected to contribute not only to PD, but also to the pathogenesis of several neurodegenerative diseases such as amyotrophic lateral sclerosis. Many studies demonstrate that excessive ROS generation during ischemia-reperfusion is responsible for the neuronal cell death in cerebral infarction. Because IPAS is effectively induced by ROS-generating intermittent hypoxia,^14^ it is possible that IPAS may be involved in neuronal degeneration. Further studies are necessary to elucidate whether IPAS has a role in the etiology of neurodegenerative disease.

## Materials and methods

### Reagents and antibodies

MG132 was obtained from Peptide Institute (Osaka, Japan), and Z-VAD-FMK was purchased from MBL (Nagoya, Japan). MPTP was obtained from Sigma-Aldrich (St Louis, MO, USA). Parkin siRNA (sc-42158) and PINK1 siRNA (sc-44598) were obtained from Santa Cruz biotechnology (Santa Cruz, CA, USA). Cycloheximide, CCCP and all other reagents were obtained from Wako Pure Chemicals (Osaka, Japan). Anti-mouse and anti-human IPAS polyclonal antibodies were produced in rabbits by immunizing with a keyhole-limpet hemocyanin-conjugated synthetic peptide corresponding to C-terminal 20 amino acids of mouse IPAS (TESSL PSWVL WALNR KNCPG) or human IPAS (NGGNC AGLGK GEGLD WPHWL), and affinity purified with peptide-conjugated cellulose. All other antibodies used were purchased from the following: anti-Myc, anti-HA (MBL); anti-FLAG (Sigma-Aldrich); anti-Tom20 (Santa Cruz biotechnology); anti-active caspase-3 (Promega, Fitchburg, WI, USA); anti-Parkin (Cell Signaling Technology, Beverly, MA, USA); anti-PINK1 (Novus Biologicals, Littleton, CO, USA); anti-TH (mouse monoclonal, Sigma-Aldrich); anti-ubiquitin—Lys48-specific, clone Apu2 and anti-ubiquitin—Lys63-specific, clone Apu3 (Merck Millipore, Darmstadt, Germany).

### Plasmid construction

Mouse IPAS cDNA was kindly provided by Dr Y Makino. pBOS-3Myc IPAS, pBOS-3Myc IPAS N, pBOS-3Myc IPAS C, pEGFP-IPAS WT, pCerulean-IPAS WT and pEGFP-IPAS deletion mutants were constructed as described.^16^ hParkin and ubiquitin cDNA were amplified from total cDNA from SH-SY5Y cells. Parkin cDNA was ligated into the EcoRV site of the pEF-BOS vector containing a 3×Myc tag or a FLAG tag. Ubiquitin cDNA was ligated into the EcoRV site of the pEF-BOS vector containing an HA tag. PINK1-pCMV6 vector (MR209092) was purchased from Origene (Rockville, MD, USA), and used after removal of the DDK-tag sequence to produce pCMV6-PINK1-Myc. pCMV6-PINK1-FLAG was constructed by replacing the Myc-DDK sequence of PINK1-pCMV6 with a FLAG tag. Introduction of point mutations was carried out by PCR using Pfu Turbo (Agilent Technologies, Santa Clara, CA, USA). The primer sequences are listed in Supplementary Table S2. Plasmids for deletion mutants of 3FLAG-hParkin were constructed by inserting appropriate PCR-amplified DNA fragments into the EcoRV site of the pBOS-3FLAG vector. All constructions were validated by sequence analysis.

### Cell culture and DNA transfection

HeLa cells and SH-SY5Y cells were obtained from the Cell Resource Center for Biomedical Research in Tohoku University and European Collection of Cell Cultures, respectively. HeLa cells and SH-SY5Y cells were maintained in EMEM (Wako) supplemented with 10% fetal bovine serum, and DMEM (Wako) with 15% fetal bovine serum and 1% non-essential amino acids, respectively. Plasmids were transfected into HeLa cells and SH-SY5Y cells using Lipofectamine 2000 and Lipofectamine LTX (Life Technologies, Carlsbad, CA, USA), respectively. Caspase inhibitor Z-VAD-FMK (10 *μ*M) was added to fresh medium 4 h after transfection to prevent cell death by IPAS expression. PINK1 and Parkin siRNA duplexes (each consisting of a pool of three target-specific sequences of 19–25 nt), were transfected into SH-SY5Y cells using Lipofectamine RNAiMAX (Life technologies).

### Fluorescence observation and immunostaining of cells

After fixation in 4% paraformaldehyde–PBS solution and permeabilization with 0.5% Triton X-100 in PBS for 5 min, cells expressing EGFP and/or Myc-tagged proteins were incubated with indicated primary antibodies at room temperature for 1 h and then with Alexa Fluor 488- or 594-conjugated secondary antibodies at room temperature for 1 h. Cells were mounted with Mowiol and examined using laser scanning confocal microscopy Olympus FV300 (Olympus, Tokyo, Japan) or fluorescence microscopy Olympus IX71 (Olympus).

### Immunoprecipitation and immunoblotting

Cells were washed with ice-cold PBS and lysed with cell lysis buffer containing 20 mM HEPES (pH 7.5), 100 mM NaCl, 1.5 mM MgCl_2_, 1 mM EGTA, 10 mM Na_2_P_2_O_7_, 10% glycerol, 1% NP-40, 1 mM dithiothreitol, 1 mM Na_3_VO_4_, 1 mM PMSF and 1% aprotinin (Sigma-Aldrich). After vortexing for 15 s, insoluble materials were removed by centrifugation. Supernatants were incubated with the indicated antibodies and protein-G Sepharose (GE Healthcare, Little Chalfont, UK) for 2 h at 4 °C, and washed three times with PBS. Proteins were released from the beads by heating at 100 °C for 3 min in 2×Laemmli sample buffer, followed by loading onto 10 or 13% SDS-polyacrylamide gels. After electrophoresis, the proteins were blotted onto a nitrocellulose membrane (GE Healthcare) and probed with indicated antibodies. After incubation with horseradish peroxidase-linked secondary antibody, blots were developed with the ECL Plus detection system (GE Healthcare). Phos-tag SDS-PAGE was performed on 8% gels containing 25.5 *μ*M Mn^2+^-phos-tag (Wako) according to the manufacturer’s protocol.

### Subcellular fractionation

HeLa and SH-SY5Y cells were frozen at −80 °C, thawed and resuspended in lysis buffer containing 20 mM HEPES (pH 7.5), 100 mM NaCl, 1.5 mM MgCl_2_, 1 mM EGTA, 10 mM Na_2_P_2_O_7_, 10% glycerol, 1 mM dithiothreitol, 1 mM Na_3_VO_4_, 1 mM PMSF and 1% aprotinin. Cell lysates were vortexed for 15 s and centrifuged at 20 400×*g* for 20 min. Pellets containing mitochondrial membranes were used as the membrane fraction, and supernatants containing soluble proteins were used as the soluble fraction. Pellets were solubilized with lysis buffer containing 1% NP-40.

### Animals

Male C57BL/6J Jms Slc mice (about 12 weeks of age) and IPAS-deficient mice were bred in a 12-h light/12-h dark cycle at 23 °C. Mice were intraperitoneally injected four times with MPTP (15 mg/kg) at 2-h intervals within a single day, and killed by inhalation of isoflurane at 3 days after MPTP treatment. Animal care and use were reviewed and approved by the Committee for Animal Research of Tohoku University.

### Construction of IPAS-Deficient Mice

The IPAS knockout mice (Accession No. CDB1169K: http://www.clst.riken.jp/arg/mutant%20mice%20list.html) were generated as described (http://www.clst.riken.jp/arg/Methods.html). Chimeric mice were intercrossed with C57BL/6J mice, and offspring mice were genotyped by Southern blotting and genomic PCR. Southern blot analysis was performed with SpeI-digested genomic DNA, and detecting a 15.6-kb band for WT and 5.6-kb and 15.6-kb bands for heterozygous mutants with the 5′ probe (569 bp). Analysis of AflII-digested genomic DNA involved detecting a 7-kb band for heterozygous mutants with the neo probe (804 bp). The probes were prepared by PCR with Prime STAR HS polymerase. Genomic PCR was performed by LA-Taq HS polymerase (Takara, Kyoto, Japan). Primers used for the reaction are described in Supplementary Table S2.

### Reverse transcription-PCR

Total RNA was extracted from mouse brain tissues using Isogen (Nippon gene, Tokyo, Japan) and cDNAs were synthesized using random hexamers and Moloney leukemia virus reverse transcriptase (Life Technologies). A fraction (1 *μ*l) of synthesized cDNA was amplified in a 20-*μ*l reaction mixture containing 5 units of Taq Hot-Start polymerase (Greiner Bio-One, Frickenhausen, Germany). PCR cycles were chosen within the linear range of amplification. The PCR procedure consisted of 18 reaction cycles for 18S rRNA, and 35 cycles for IPAS and HIF-3*α*: 95 °C for 30 s, 60 °C for 30 s and 72 °C for 30 s. The bands were quantified using ImageJ software (National Institutes of Health). The primers used for the reactions are shown in Supplementary Table S2.

### Immunohistochemistry

The brain samples were fixed in neutral-buffered formalin (Mildform10N, Wako) at 4 °C overnight, dehydrated in an alcohol series, embedded in paraffin, and serially sectioned at 8 *μ*m throughout SNpc. Every third section of each brain was deparaffinized in xylene, rehydrated through a graded series of ethanol, and incubated with 5% goat serum in PBS for 1 h. Anti-TH (1:2000) and anti-mIPAS (1:200) were used as the primary antibodies. For anti-TH antibody-treated samples, sections were incubated in HistoVT One (Nacalai Tesque, Kyoto, Japan) at 90 °C for 20 min before blocking. The number of TH-positive neurons in each section was added to provide a measure of the total number of SNpc TH-positive neurons for each mouse.

### Human subjects

Twelve autopsy cases (Supplementary Table S1: six sporadic PD and six controls) were used in this study. All diagnoses were confirmed by neuropathological examination. The institutional ethics committee of University of Niigata Faculty of Medicine approved the usage of human subjects in this study. Written informed consent for autopsy, collection of samples and subsequent analysis was obtained from the next of kin of all the deceased involved in this study.

### Statistical analysis

Data are given as mean±S.D., with the number of experiments indicated. Statistical significance was established at *P*<0.05 by unpaired *t*-tests. The Tukey–Kramer *post hoc* test following two-way analysis of variance was used to detect statistical significance between number of TH-positive neurons in MPTP-treated WT and IPAS-deficient mice.

## Abbreviations

CCCP: carbonyl cyanide m-chlorophenyl hydrazone
Co-IP: co-immunoprecipitation
HIF: hypoxia-inducible factor
IPAS: Inhibitory PAS domain protein
MPTP: 1-methyl-4-phenyl-1,2,3,6-tetrahydropyridine
PD: Parkinson's disease
PINK1: PTEN-induced putative kinase 1
ROS: reactive oxygen species
SNpc: substantia nigra pars compacta
TH: tyrosine hydroxylase
MPP^+^: 1-methyl-4-phenylpyridinium
hParkin: human Parkin
mParkin: mouse Parkin
WT: wild type.
