# Supplementary material for: Involvement of inhibitory PAS domain protein in neuronal cell death in Parkinson’s disease
Source: Cell Death Discov. 2015 Aug 17;1:15015–. doi: 10.1038/cddiscovery.2015.15 (PMC4981001; doi:10.1038/cddiscovery.2015.15)
Supplement: Supplementary Information [file cddiscovery201515-s1.doc]

**Legends for Supplementary Figures**

**Supplementary Figure 1. Polyubiquitination of IPAS by Parkin.** (**a** and **b**) Interaction between IPAS and Parkin by MG132 or CCCP treatment. HeLa cells were transfected with expression plasmids of Myc-IPAS and FLAG-mParkin, treated with MG132 (**a**) or CCCP (**b**), and analyzed by immunoprecipitation with antibody against FLAG followed by immunoblotting with antibody against Myc. (**c**) Binding of IPAS to mParkin by CCCP and MG132 treatment. HeLa cells (upper panel) and SH-SY5Y cells (lower panel) were transfected with expression plasmids of Myc-IPAS and FLAG-mParkin, treated with CCCP and MG132, and analyzed as shown in (**a**). (**d**) Polyubiquitination of IPAS by mParkin through K48 linkage. HeLa cells were transfected with indicated combinations of expression plasmids, treated with or without MG132, and analyzed by immunoprecipitation with antibody against Myc followed by immunoblotting with antibody against HA, Ub-Lys48, or Ub-Lys63.

**Supplementary Figure 2. Interaction between IPAS deletion mutants and mParkin.** (**a**) Schematic representation of the domain structure of IPAS and its deletion mutants. bHLH, basic helix-loop-helix motif. (**b**) Binding of IPAS to Parkin through its C-terminal region. HeLa cells were transfected with plasmids coding for IPAS WT, IPAS N or IPAS C together with pFLAG-mParkin. Co-IP assays were carried out as in Supplementary Fig. 1a. ns, nonspecific bands. (**c**) Colocalization of IPAS WT and IPAS C with Parkin. SH-SY5Y cells were transfected with pEGFP-IPAS WT, pEGFP-IPAS N or pEGFP-IPAS C together with pMyc-mParkin, and treated with CCCP and MG132. Cells were stained with anti-Parkin antibody. The subcellular localization of IPAS WT, IPAS deletion mutants and Parkin was observed using a confocal microscope.

**Supplementary Figure 3. Stabilization of PINK1 by CCCP treatment.** (**a**) Distribution of marker proteins in membrane and soluble fractions. Soluble and membrane fractions were separated as described in “Experimental procedures”. Marker proteins were detected by immunoblotting. (**b** and **c**) Induction of PINK1 by the CCCP and/or MG132 treatment. HeLa cells (**b**) and SH-SY5Y cells (**c**) were transfected with a PINK1-FLAG expression plasmid and treated with CCCP and/or MG132 as indicated. After separation into membrane and soluble fractions, each fraction was analyzed by immunoblotting using the anti-FLAG antibody.

**Supplementary Figure 4. Co-localization of EGFP-IPAS WT and C with PINK1.** SH-SY5Y cells were transfected with plasmids of EGFP-IPAS WT, IPAS N and IPAS C together with pPINK1-Myc. After immunostaining with anti-Myc antibody, cells were observed with a confocal microscope.

**Supplementary Figure 5. Protection of IPAS-induced apoptosis by coexpression of Parkin.** SH-SY5Y cells were transfected with Cerulean-IPAS and Myc-mParkin WT, Myc-hParkin WT or Myc-Parkin T415N. 24 h after transfection activation of caspase-3 were examined by an antibody to active caspase-3. Representative images were shown of 3 independent experiments.

**Supplementary Figure 6. Induction of IPAS by MPTP and hypoxia.** (**a**) Induction of IPAS mRNA in the cerebrum (left) and cerebellum (right) by intraperitoneal injection of MPTP. The PCR products were analyzed on a 2% agarose gel. Representative images of 3 independent experiments were shown. Each band was quantified by using ImageJ software. Data shown in bar graphs are mean ± SD of three independent experiments (lower). (**b**) Induction of IPAS in the cerebellum by the exposure of mice to hypoxia. Mice were exposed to 6% hypoxia for indicated time period and dissected. IPAS mRNA in the cerebellum was determined by RT-PCR as in (**a**). IPAS protein expression was examined by immunohistochemistry using the antibody against IPAS. A weak normoxic expression was detected in the Purkinje cells as described by Makino et al. (2001), and the expression was augmented in hypoxia. Arrows indicate IPAS-positive Purkinje cells. * p<0.05, ** p<0.01.

**Supplementary Figure 7. Strategy for production of IPAS-deficient mice.** (**a**) Schematic representation of IPAS exon16-targeted disruption strategy. Partial restriction map of the HIF-3α locus (top), gene-targeting vector (middle), and the expected structure of the mutated locus (bottom) are shown. Two filled boxes on the Wt allele represent exon 15 and intron 15 (left) and 3’ untranslated region of exon 16 (right). The open box between the filled boxes denotes the protein-coding region plus 90-bp 3’ untranslated region encoded by exon 16. Neo; neomycin cassette. The neomycin cassette is flanked by loxP sites (solid triangles). (**b**) Genomic PCR and Southern blot analyses of IPAS-deficient mice. Representative PCR analysis of genomic DNA for the HIF-3α gene and neomycin cassette (left). Primer positions are indicated by arrows in (**a**). Southern blot analysis of mouse tail genomic DNA digested with Spe I (center) and Afl II (right). The positions of 5’ probe and Neo probe are indicated in (**a**) as labeled “probe” in the top (5’ probe) and middle (Neo probe) map, respectively, along with expected sizes of hybridized fragments.
