# Supplementary figures and images for: Involvement of inhibitory PAS domain protein in neuronal cell death in Parkinson’s disease
Source: Cell Death Discov. 2015 Aug 17;1:15015–. doi: 10.1038/cddiscovery.2015.15 (PMC4981001; doi:10.1038/cddiscovery.2015.15)

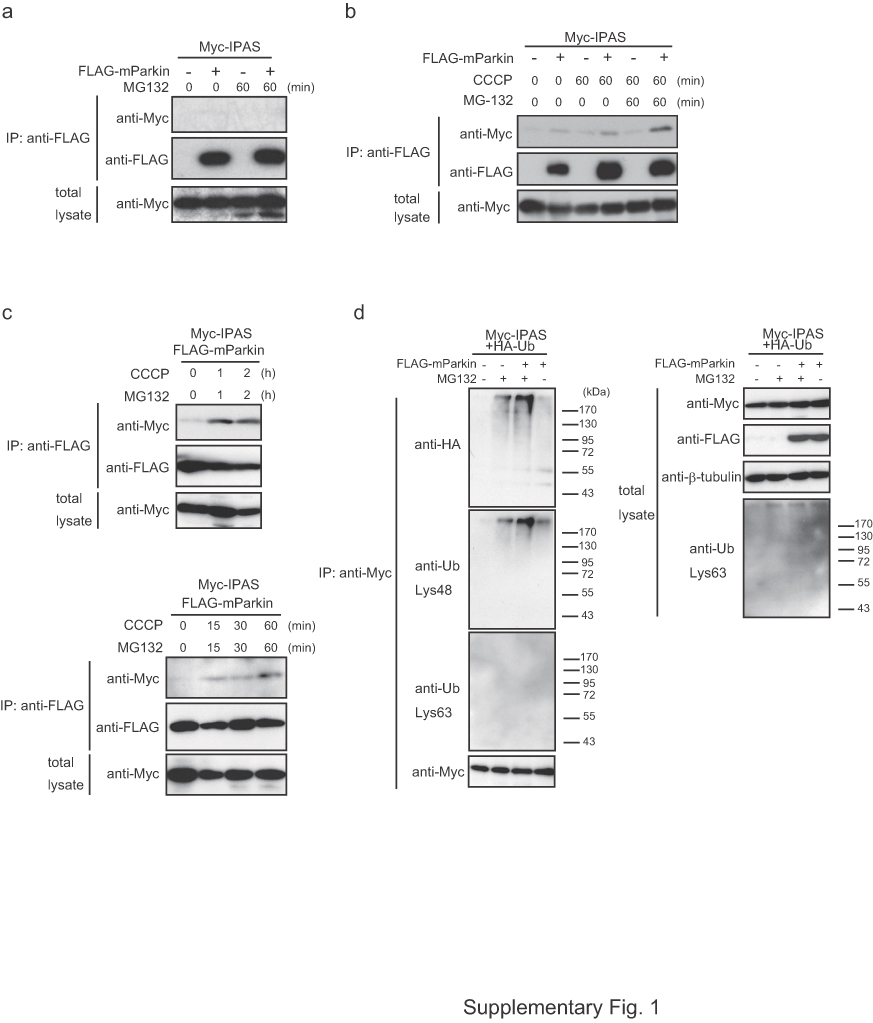

Supplement: Supplementary Figure S1 [file cddiscovery201515-s2.jpg]

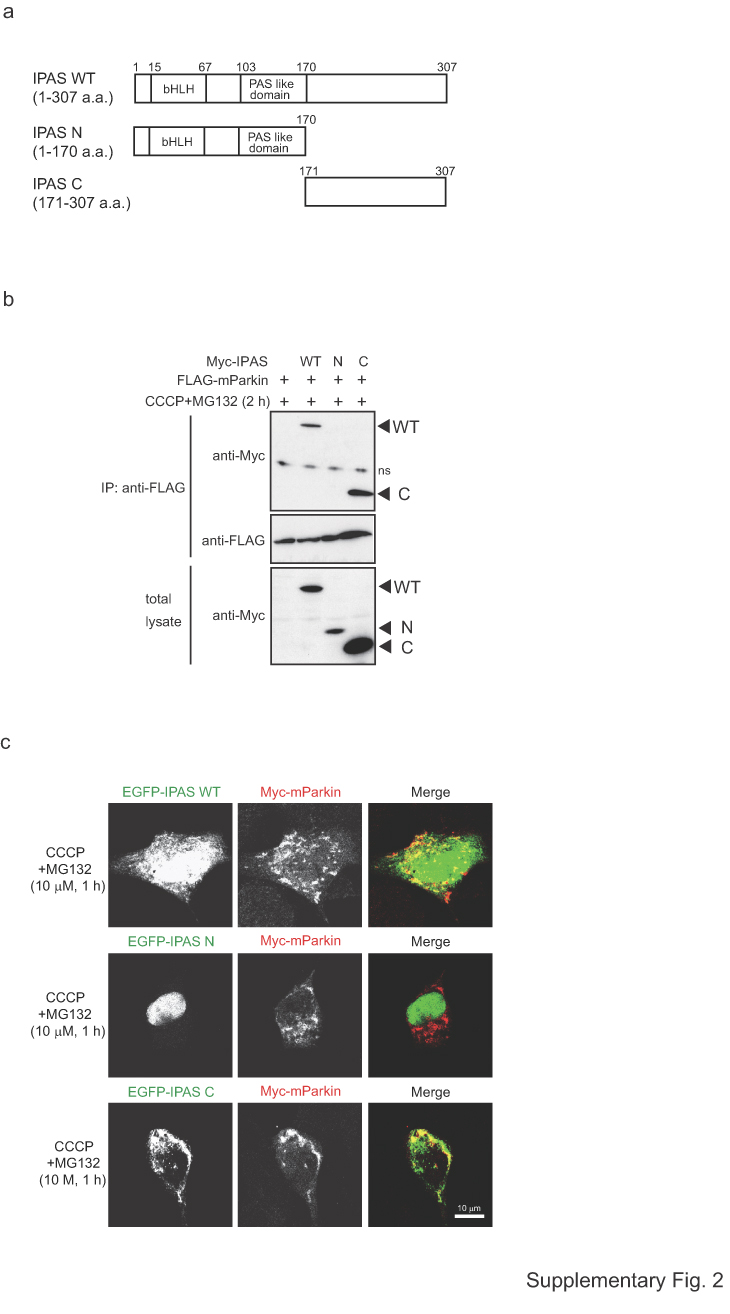

Supplement: Supplementary Figure S2 [file cddiscovery201515-s3.jpg]

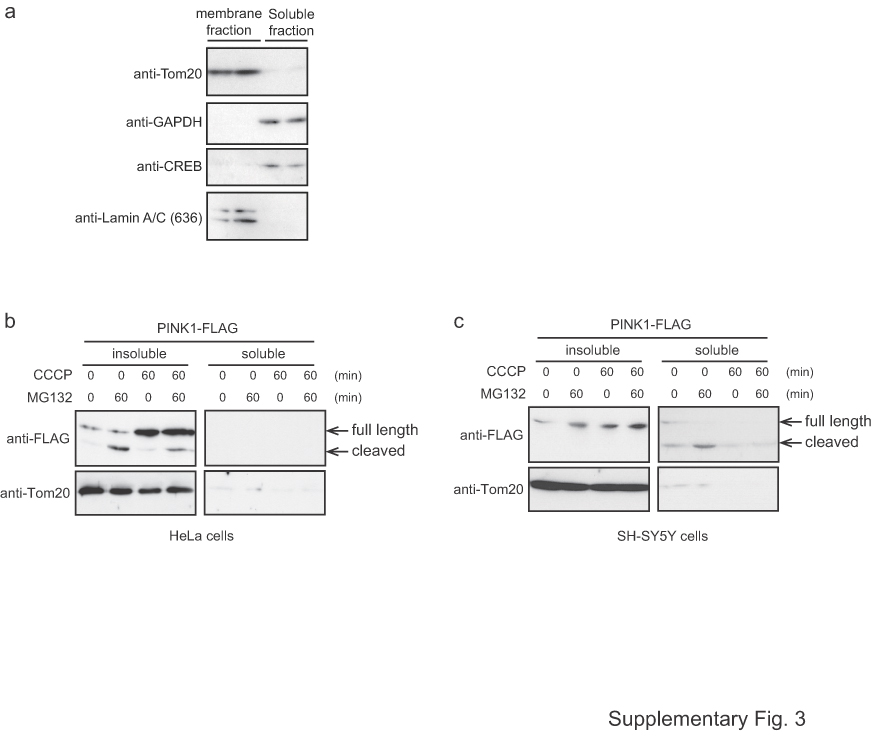

Supplement: Supplementary Figure S3 [file cddiscovery201515-s4.jpg]

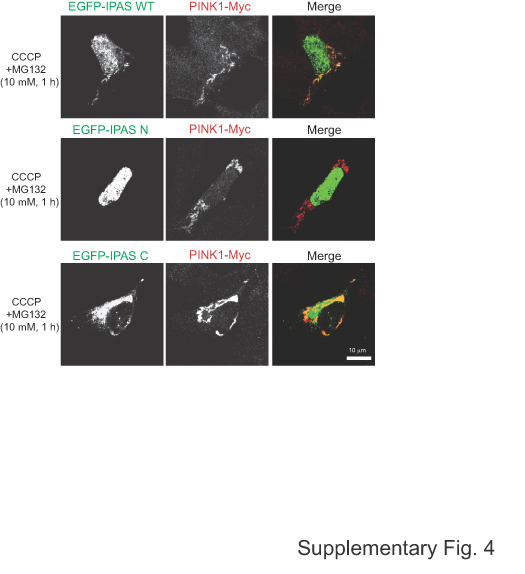

Supplement: Supplementary Figure S4 [file cddiscovery201515-s5.jpg]

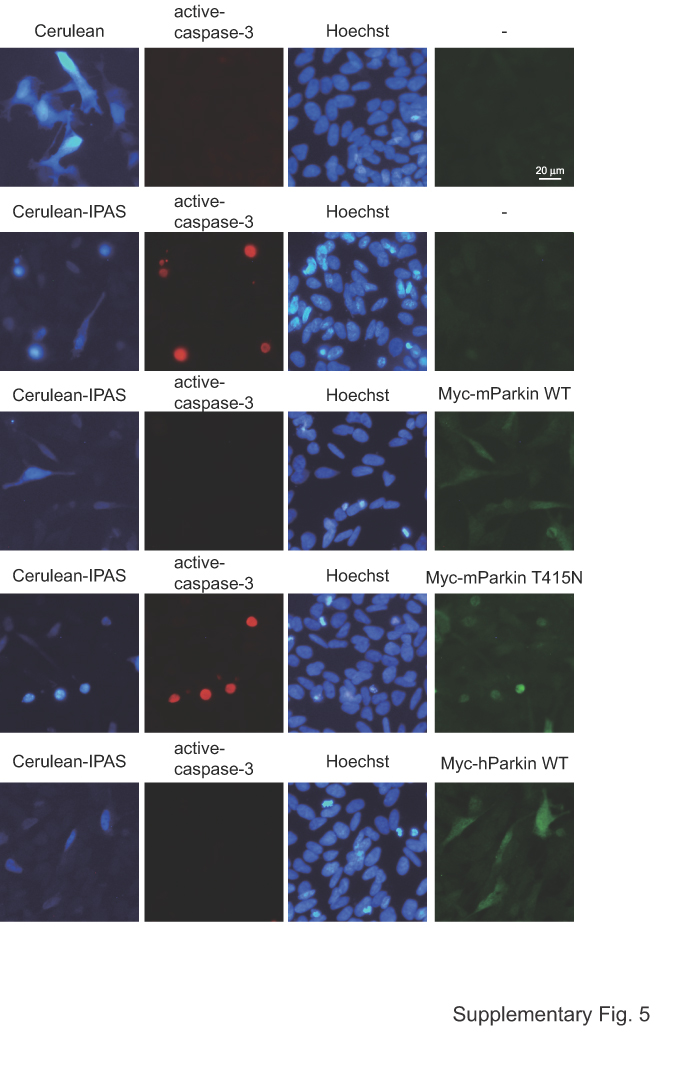

Supplement: Supplementary Figure S5 [file cddiscovery201515-s6.jpg]

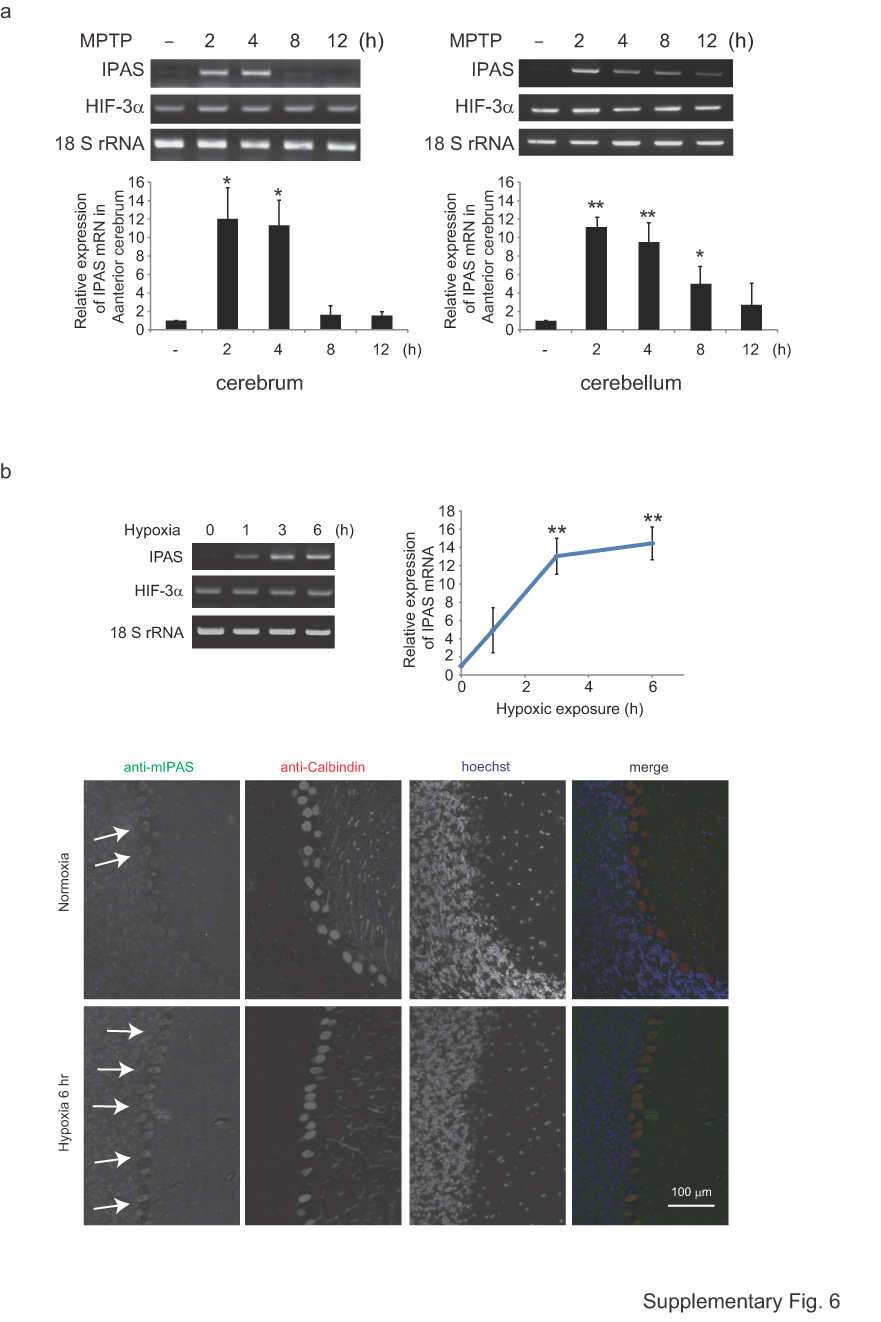

Supplement: Supplementary Figure S6 [file cddiscovery201515-s7.jpg]

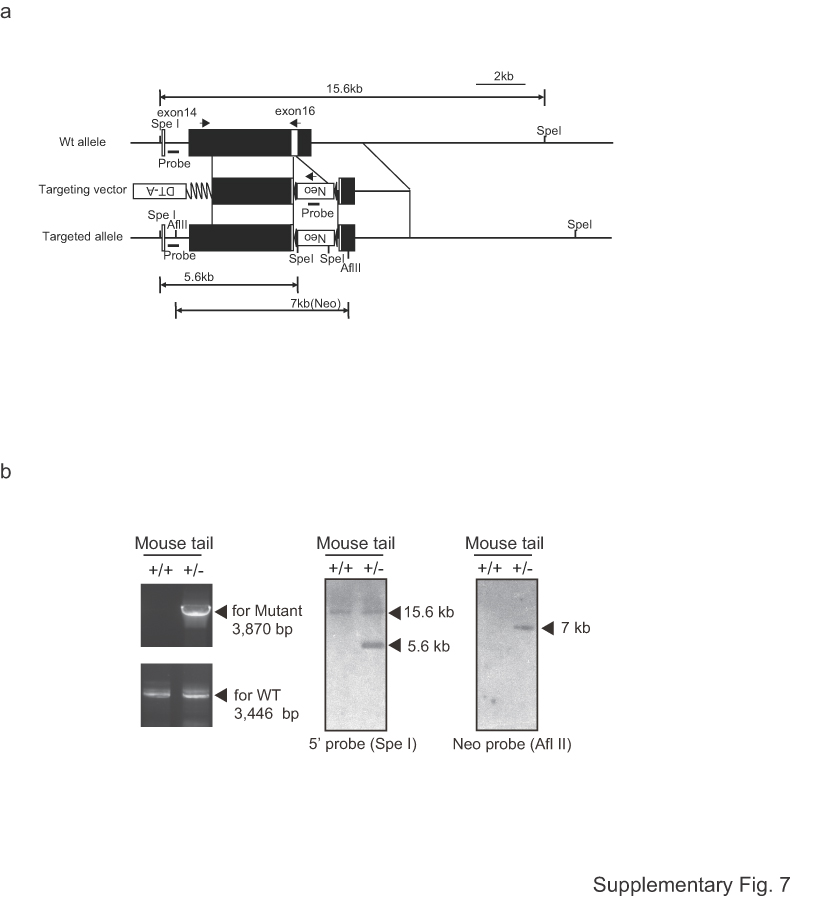

Supplement: Supplementary Figure S7 [file cddiscovery201515-s8.jpg]
